# Regional Differences in Lung Ventilation During the Early Transition Period in Late Preterm and Term Neonates Assessed by Electrical Impedance Tomography

**DOI:** 10.3390/children11111314

**Published:** 2024-10-29

**Authors:** Adomas Janulionis, Viktorija Sutova, Vita Langiene, Ernestas Virsilas, Violeta Drejeriene, Arunas Liubsys, Arunas Valiulis

**Affiliations:** 1 Clinic of Children’s Diseases, Institute of Clinical Medicine, Medical Faculty of Vilnius University, M. K. Čiurlionio 21, 03101 Vilnius, Lithuania; viktorija.sutova@santa.lt (V.S.); arunas.liubsys@santa.lt (A.L.); arunas.valiulis@mf.vu.lt (A.V.); 2 Department of Obstetrics and Gynaecology, Vilnius City Clinical Hospital, Antakalnio 57, 10207 Vilnius, Lithuania; 3 Centre of Neonatology, Vilnius University Hospital Santaros Clinics, Antariškių 2, 08661 Vilnius, Lithuania

**Keywords:** lung ventilation, early adaptation, electrical impedance tomography, term neonates, late preterm neonates

## Abstract

Background: Changes in lung ventilation are well documented in term neonates while in late preterm neonates these patterns are poorly understood despite their increased risk of respiratory morbidity. Objectives: The study aimed to compare and clarify the differences in regional lung ventilation of late preterm and term neonates during the early adaptation period using electrical impedance tomography (EIT). Material and methods: The case-control study was conducted in the years 2020–2022. It included 51 late preterm neonates (LPN, Study group) and 45 term neonates (TN, Control) born by normal vaginal delivery (NVD). EIT examinations were performed with a Swisstom BB2 (Switzerland) equipment. The data recordings were performed no later than 30 (I Record), 60 (II), and 90 (III) minutes after the birth. Results: Statistically significant differences between LPN and TN were observed in the non-dependent lung areas at I record, with more silent spaces observed in the LPN (*p* < 0.001). Differences in the dependent lung regions were observed across all recordings, with LPN demonstrating more silent spaces (*p* < 0.001). LPN demonstrated greater stretch-related changes in the 10% and 20% stretch categories across all recordings, while TN showed greater changes in the 50%, 70%, and 90% categories. Tidal volumes in the right lung of TN are distributed more towards the ventral and central ventral regions. In contrast, tidal volumes of LPN are distributed to the central dorsal and dorsal regions of the right lung. Conclusions: LPN during the first 90 min after the birth show reduced lung ventilation assessed by EIT, suggesting a possible impairment of early postnatal adaptation.

## 1. Introduction

The period of transition from intrauterine to extrauterine life is critical to ensure effective pulmonary function in neonates. The early period of neonatal life is marked by rapid and extreme changes in lung ventilation as it transitions from a fluid-filled organ to one filled with air in volumes capable of efficient gas exchange. While this process has been relatively well documented in term neonates (TNs), the pulmonary mechanics of late preterm neonates (LPNs)—born at 34 to 36 weeks of gestation—are poorly understood, despite their increased risk of respiratory morbidity [1,2]. Understanding the patterns of lung ventilation in term and late preterm neonates in this time is crucial for the further improvement of neonatal care.

Electrical impedance tomography (EIT) is a recent tool for the instantaneous monitoring of regional pulmonary ventilation in neonates [3,4,5,6]. EIT is a non-invasive method based on thoracic impedance variation [7,8,9,10]. Studies using EIT in term infants explore the aspects concerning the regulation of lung ventilation during the early postnatal period [11]. However, there is little research comparing the patterns of pulmonary ventilation in term and late preterm neonates using EIT.

LPNs are a group of newborns with characteristics that set them apart from full-term neonates. This distinction may raise doubts, as LPNs are considered close to term. However, despite their proximity to term, LPNs are at a higher risk of experiencing conditions like respiratory distress syndrome, transient neonatal tachypnea, and other respiratory illnesses [12,13,14,15,16]. These conditions arise from lung immaturity as well as the delayed production of pulmonary surfactant, resulting in four times higher mortality [17]. Therefore, analyzing the dynamics of pulmonary ventilation in late preterm neonates in the period of early postnatal adaptation can improve the quality of care for this vulnerable group of neonates.

The study aimed to compare the regional lung ventilation of late preterm and term neonates during the early adaptation period using EIT.

## 2. Materials and Methods

### 2.1. Subjects

**Scheme 1:**
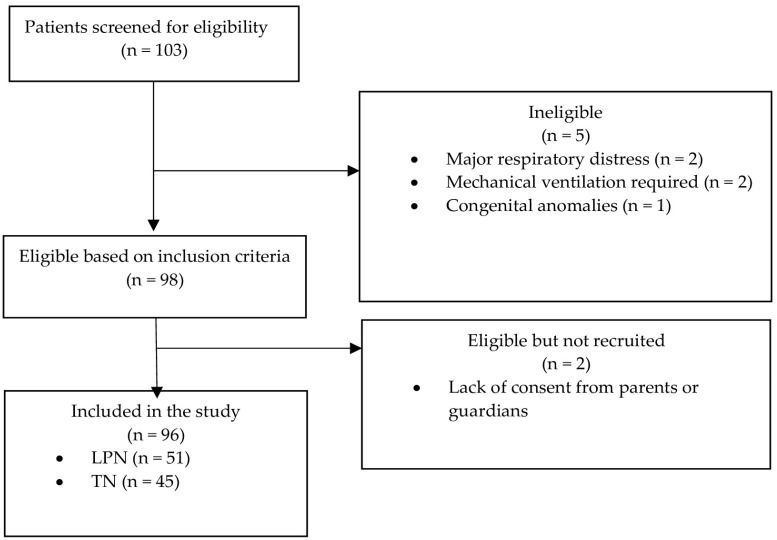
STROBE participant flow diagram.

Our case-control study included 51 late preterm spontaneously breathing neonates (study group) and 45 term spontaneously breathing neonates (control group) born at Vilnius City Clinical Hospital, Department of Obstetrics and Gynaecology in the years 2020–2022 (Scheme 1). The neonates were systematically selected based on predetermined inclusion criteria: (1) gestational age: term neonates—between 37 and 42 weeks of gestation, late preterm neonates—between 34 and 36 weeks of gestation; (2) spontaneous breathing, without the need for respiratory support or mechanical ventilation; (3) stable health condition; (4) consent from parents or guardians. The exclusion criteria were as follows: (1) clear signs of respiratory distress (e.g., grunting, nasal flaring, chest retractions); (2) clinical or radiological signs of respiratory pathology (e.g., respiratory distress syndrome, pneumothorax, persistent pulmonary hypertension, meconium aspiration syndrome); (3) diagnosis of congenital respiratory system disease (e.g., cystic lung disease, congenital pneumonia); (4) diagnosis of congenital pathology or chromosomal anomaly affecting other organs; (5) complications during delivery (e.g., severe asphyxia) that required resuscitation; (6) lack of consent from parents or guardians.

The study was approved by the Vilnius Region Biomedical Research Ethics Committee (No 1287, 24 November 2021), and written informed consent was obtained from the parents of the neonates.

**Figure 1:**
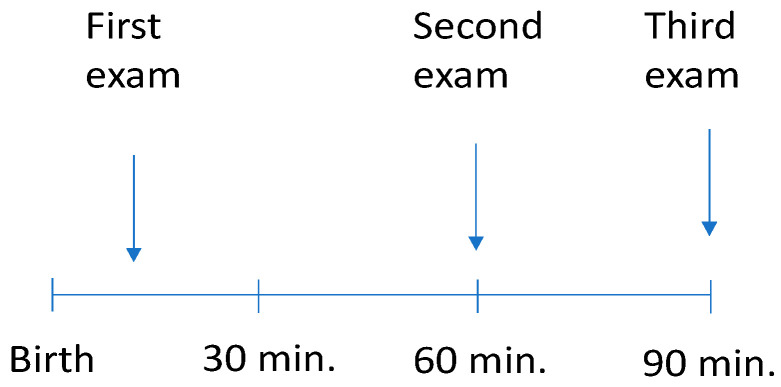
EIT data recording intervals and their timing.

The data collection utilized the electrical impedance tomography device (Swisstom BB2, Landquart, Switzerland). A NEO SensorBelt equipped with integrated 32 electrodes was positioned circumferentially around the thorax at the nipple level, operating at a sample rate of 47.68 Hz. Three data recording sessions were conducted on the day of birth for each neonate. Data were collected at three intervals: immediately after birth (0 min), 60 min after birth, and 90 min after birth, respectively (Figure 1).

All recordings were conducted while the infants were in the supine position to standardize the measurements and minimize variability in lung ventilation distribution caused by gravitational effects. It ensures uniformity across participants, reducing the influence of different body positions on the EIT results. This allowed for a consistent and comparable assessment of lung ventilation patterns between the LPNs and TNs. For breastfeeding sessions, measurements were delayed to avoid disrupting mother–infant bonding. Similarly, for any procedures such as blood draws, heel pricks, or routine nursing care, measurements were postponed from the scheduled time frames unless the delay exceeded 30 min beyond the designated time frame. Each individual EIT recording comprised a minimum of 5 min of data collection. All of the studied infants were spontaneously breathing.

The data were analyzed using Ibex 1.4 software. The impedance signal was extracted using a respiratory rate filtering function. The following EIT derivatives were compared between the two groups of subjects:

1. The center of ventilation (CoV), describing the best-ventilated lung areas;
2. Silent spaces, describing lung *areas* that receive *minimal* or *no ventilation* (impedance changes <10%);
3. Relative stretch, indicating the potential of the lungs to expand during inspiration (monitoring ventilation-related changes in electrical impedance and conductivity, and creating images of local ventilation distribution);
4. Relative tidal volume, divided by tenths of total ventilated lung area (where each tenth represents 10% of the total ventilated area);
5. Parts of tidal volume in the right and left lungs separately, referring to the sum of tidal volumes per lung and regional distribution: it involves segmenting the lung into regions (ventral, dorsal, central ventral, central dorsal) based on the cross-sectional images. This segmentation is generally done in a manner that reflects the anatomical and functional regions of interest. For many clinical and research applications, focusing on these predefined regions provides sufficient information about ventilation distribution and dynamics.

### 2.2. Statistical Analysis

The results were analyzed with SPSS statistical software, version 22. The Mann–Whitney statistical criterion was used to compare the results between groups. The results of the statistical analysis were considered statistically significant if the calculated p-value was less than the 0.05 level of significance (*p* < 0.05).

## 3. Results

Table 1 provides a comparison of the main characteristics between the study group (late preterm neonates, n = 51) and the control group (term neonates, n = 45). The variables listed include the following: (1) Gestational age: the average gestational age of LPNs was 35 weeks, while the average gestational age of TNs was 39 weeks; (2) Maturity index: the LPNs had a lower maturity index compared to the TNs group; (3) Birth weight: the LPNs had a lower average birth weight compared to term neonates (2756 g vs. 3464 g); (4) Cord blood pH: both groups showed similar cord blood pH levels, indicating comparable acid-base status at birth; (5) Apgar scores: both groups had similar Apgar scores at 1 min and 5 min, showing overall stable neonatal conditions.

The first data recording was performed as early as possible after birth, depending on the condition of the newborn, but no later than within 30 min. (within 13 min. on average) after birth. The second data recording took place at 62 min. on average and the third one at 1.5 h (93 min on average) after birth.

### 3.1. Silent Spaces

Statistically significant differences between late preterm and term neonates were observed in the non-dependent lung areas (anterior or less influenced by gravity) at the time of the first recording, with more silent spaces observed in the late preterm neonates group (*p* < 0.001). Statistically significant differences between the groups were consistently observed in the dependent lung regions (posterior or more affected by gravity) in all three recordings, with the late preterm neonates group demonstrating more silent spaces (*p* < 0.001 in all three recordings) (Table 2, Table 3 and Table 4).

### 3.2. Relative Stretch of Lung Tissue

There were no statistically significant differences in the quartile (−) (the relative stretch values in the lower quartile) and median (the middle 50% of the relative stretch values) between preterm and term infants (*p* > 0.05). A statistically significant difference of the quartile (+) (the relative stretch values in the upper quartile) values between late preterm and term neonates was observed at record III, and greater stretch-related changes were observed in the term neonates group (*p* < 0.05) (Table 5).

Late preterm neonates had greater stretch-related changes than term neonates in the 10% and 20% relative stretch categories in all three records (i.e., smaller changes in the stretch; gray spaces occupy a larger proportion of the lung image in the study group), with *p* < 0.001 and *p* = 0.001, respectively, at record I; *p* < 0.001 and *p* = 0.039, respectively, at record II; *p* < 0.001 and *p* < 0.001 respectively at record III. Conversely, term neonates had greater stretch-related changes than late preterm neonates in the 70% category at record I (*p* = 0.012) and the 50% and 90% categories at record II (*p* = 0.039 and *p* = 0.005, respectively) (Table 6, Table 7 and Table 8).

### 3.3. Center of Ventilation

Statistically significant differences related to this parameter were visible in all three records in the ventral-dorsal projection (*p* < 0.001, *p* < 0.001, *p* < 0.001). Late preterm neonates showed higher ventilation rates toward the ventral (anterior) side of the lungs than term neonates. There were no statistically significant differences in the right-left projection of ventilation in late preterm and term neonates (*p* > 0.05) (Table 9, Table 10 and Table 11). 

### 3.4. Distribution of Tidal Volume

There was no significant temporal variation in the tidal volume distribution in the left and right lungs between the study and control cohorts (*p* > 0.05). However, significant differences were observed in the distribution of right lung volumes in all three records (*p* < 0.01). Compared to late preterm neonates, tidal volumes in the right lung of term neonates are distributed more towards the ventral and central-ventral regions (record I: *p* < 0.001, record II: *p* < 0.001 *p* = 0.001, record III: *p* < 0.001, *p* = 0.001). In contrast, the tidal volume of late preterm neonates was distributed to the central-dorsal and dorsal regions of the right lung compared to term neonates (*p* < 0.001 at records I and II; *p* = 0.006 and *p* < 0.001 respectively at record III). All three records showed significant differences in the distribution of left lung tidal volumes between term and late preterm neonates in different areas. Generally, in term neonates, tidal volume was distributed towards the ventral regions of the left lung (*p* < 0.001 in records I and II; *p* = 0.001 in record III), whereas, in late preterm neonates, tidal volume distribution was towards the central-dorsal region (*p* < 0.001). In addition, it was observed that the central-ventral and dorsal regions of the left lung differed, although not consistently, between the groups. At record III, in late preterm neonates, tidal volumes were distributed to the central-ventral regions of the left lung (*p* = 0.046). At record I, tidal volumes were distributed in this group to the dorsal regions of the left lung (*p* = 0.023) (Table 12, Table 13 and Table 14).

## 4. Discussion

Our study revealed lower lung ventilation in late preterm neonates compared to term neonates. This means that late preterm neonates are at higher risk of having inadequately aerated areas in the lungs during the adaptation period, which may lead to respiratory complications later on. LPNs are at a developmental stage where critical processes, such as alveolarization and surfactant production, remain incomplete. These neonates have not yet achieved full functional maturity of the lungs, resulting in a diminished capacity for effective alveolar fluid clearance and lung expansion. The interstitial matrix, which plays a key role in supporting the structural integrity of the alveoli, is also underdeveloped, contributing to reduced regional lung compliance. Consequently, ventral-dorsal and right-left ventilation disparities are observed, particularly in the basal lung regions, as these areas are more prone to atelectasis and inadequate ventilation in the absence of mature alveolar surfactant (minimal surfactant protein B expression, etc.). Moreover, the absence of sufficient active mechanisms for alveolar fluid reabsorption exacerbates the impaired gas exchange observed in LPNs. These physiopathological factors contribute to the reduced ability of the lungs to expand evenly across regions, which is reflected in the EIT data. The lack of surfactant not only disrupts alveolar stability but also worsens ventilation-perfusion mismatch, leading to greater reliance on mechanical assistance to maintain adequate oxygenation. The preterm transition may be the cause of respiratory distress syndrome (RDS), transient tachypnea of the newborn (TTNs), and other respiratory diseases [18]. Kalyoncu et al. reported that late preterm neonates have a higher risk of respiratory distress compared to term neonates. The most common conditions observed were transient tachypnea of the newborn, then respiratory distress syndrome (RDS), and pneumonia [19]. Atasay et al.’s study revealed that 30% of LPNs included experienced respiratory distress [20]. The functional challenges faced by LPNs include difficulties in maintaining functional residual lung capacity, increased airway resistance, and risk of airway collapse. Additionally, the transition from lung fluid to breathing ambient air is crucial but could be delayed in preterm births due to underdeveloped epithelial sodium channels, especially without the onset of spontaneous labor [21]. Environmental factors such as maternal smoking, air pollution, and pregnancy-related stress may worsen respiratory problems in this group [22]. Studies suggest that adapted respiratory support strategies are necessary to reduce the risk of RDS and other complications linked to lung immaturity in late preterm infants. As dependent lung regions are usually posterior, depending on the position of the neonate, they may be more exposed to gravity and have lower ventilation compared to non-dependent regions. The higher number of “silent” spaces in the dependent lung regions of LPNs suggests that these areas could have impaired ventilation/aeration or overdistension, which may lead to a mismatch between ventilation and blood flow and may impair respiratory function. This highlights the importance of close monitoring to ensure adequate ventilation and optimal pulmonary blood flow and to predict respiratory outcomes during early adaptation. The reduced ventilation, especially in the dependent areas of the lungs, aligns with the observed need for specific volume and pressure strategies during resuscitation emphasized in the study by van Vonderen et al., which presented a broad characterization of neonatal transition [23]. While optimizing the posture may improve respiratory function, evaluated by respiratory inductive plethysmography, as per Gouna et al., EIT could potentially serve as a sensitive method to detect basic pulmonary adaptation challenges in LPNs as early as 30 min post-birth. This convergence of findings implies the possibility of integrating both positional therapy and EIT monitoring in the clinical management of late preterm infants to potentially improve respiratory outcomes [24].

Differences in relative stretch and regional ventilation distribution patterns were found between the study and control groups. The increased ventilation distribution towards the ventral (anterior) part of the lung in LPNs probably indicates differences in lung development and breathing mechanics between the two groups. While the overall lung volume distribution in each lung remains relatively consistent over time, there has been a variation in lung volume distribution between the study and control groups, especially in the right lung. Healthcare providers should consider these distinctions when administering support to term and late preterm neonates to prevent issues such as atelectasis or lung injury. Relative stretch is the potential for lung tissue to expand with air intake. It reflects the flexibility or elasticity of the lung tissue and is an important factor in determining lung function. The absence of statistically significant differences in the relative stretch between the quartiles (−) and medians of the two groups of neonates suggests that, overall, the initial lung compliance of the two groups during the adaptation period may be similar. However, the statistically significant differences observed between the quartile (+) values at record III suggest that under certain conditions or at certain stages of lung adaptation, late preterm and term neonates may have different lung compliance levels. The higher relative stretch quartile (+) value observed in the term neonates group at record III suggests that term neonates may have better lung compliance or elasticity in response to increased ventilatory demand at this stage compared to late preterm neonates. LPNs exhibit a higher level of relative stretch in the lower percentage ranges, suggesting differences in lung maturity and flexibility when compared to term neonates. The greater proportion of lung tissue with lower levels of expansion in this group highlights the importance of close monitoring and care to ensure optimal lung aeration and gas exchange, especially during ongoing lung development. On the other hand, the higher stretch level in the higher percentage ranges among term neonates may indicate a better expansion of lung tissue, possibly attributable to increased air intake and a mature respiratory system, resulting in better respiratory function and adaptation after birth. However, in some cases, this could indicate repetitive opening and collapsing of lung areas, which is not necessarily beneficial.

There are a large number of studies assessing the respiratory status and management strategies of preterm and term neonates. Therefore, the comparison of lung ventilation in newborns during the early adaptation period, using electrical impedance tomography, is of great scientific interest. Many studies were focused on very preterm neonates [25,26,27,28,29]. While we found that late preterm neonates exhibited lower lung ventilation compared to term neonates, indicating a higher risk of respiratory complications, Gaertner et al. demonstrated the predictive value of EIT parameters for respiratory outcomes in very preterm neonates during the early adaptation period. Analyzing EIT parameters recorded as early as 30 min after birth, the investigators found certain markers of lung aeration, namely a lower percentage of aerated lung volume and a higher aeration homogeneity ratio, which accurately predicted the need for oxygen therapy 28 days after birth. This suggests that EIT may be a useful tool to adapt individual respiratory support strategies for this population and improve outcomes. However, the study has some limitations, such as the small sample size. Moreover, larger prospective studies are needed to confirm these results. Nevertheless, the results point to the need for early screening of lung function in preterm neonates, and in this context, the EIT has a promising role to play. This opens the door for further research and potential clinical application to optimize respiratory care in this vulnerable population [30]. The research conducted by Bentsen et al. presents longitudinal data on lung function in extremely preterm neonates at birth, which is important for putting our own study results into context. While Bentsen et al.’s study monitored a group of infants over time to evaluate lung function closer to birth, our study specifically focuses on the period after delivery using EIT to track changes in lung ventilation within the first 90 min post birth. Our findings show differences in lung ventilation between LPNs and TNs, with LPNs exhibiting reduced lung ventilation during the early adaptation period. The use of EIT allows us to observe these differences in real time, potentially assisting clinicians with prompt decision-making. In contrast, Bentsen et al.’s study employed electromagnetic inductance plethysmography to understand lung function in preterm infants at a later stage of development providing insights into respiratory maturation over time. Nevertheless, our study highlights the nature of the immediate postnatal period by demonstrating compromised lung ventilation in LPNs, suggesting that early adaptation challenges may stem from incomplete structural and functional maturity of the lungs. While EIT enables real-time assessments, plethysmography offers measurements of thoracic gas volume and functional residual capacity but lacks immediate bedside applicability and temporal resolution. Both EIT and electromagnetic inductance plethysmography work together to enhance our knowledge of newborn lung adaptation and help to improve care in intensive units for better support of LPNs [31]. In addition, Veneroni et al. conducted a study using the forced oscillation technique (FOT) to monitor real-time lung aeration and mechanics in preterm neonates receiving respiratory support [32]. Their findings revealed variability in initial lung aeration, impacting the effectiveness of subsequent respiratory interventions. Just like our research, they highlighted the heterogeneity in lung aeration among preterm infants at birth, as well as emphasized the importance of diagnostic approaches to guide respiratory care. There is an agreement on the critical role of lung volume recruitment soon after birth. While Veneroni et al. focused on the ability of FOT to monitor changes in respiratory mechanics during mechanical ventilation, our research using EIT offered a detailed view of the spatial distribution of lung ventilation, highlighting changes in actual lung volume and potential regional vulnerability. Tana et al. conducted a study focusing on preterm neonates with respiratory distress syndrome and analyzed lung volume changes and hemodynamic status during high-frequency ventilation [33]. In contrast, our research focused on a group of late preterm and term neonates born via normal vaginal delivery who were assessed under spontaneous breathing conditions. Our study findings support Tana et al.’s claim regarding the vulnerability of lung function in preterm neonates. However, we expand on this by examining late preterm neonates. In our research, we used EIT to demonstrate how late preterm babies may have lower lung ventilation as seen in the distinct ventilation patterns across different regions of their lungs. This indicates a vulnerability to respiratory complications. Our results align with Tana et al.’s findings on preterm infants with respiratory distress syndrome and suggest that even without obvious illness late preterm neonates exhibit noticeable variations in lung function that could make them more susceptible to respiratory issues.

Furthermore, studies highlight the vulnerability of late preterm neonates, often referred to as “near-term”, due to their immature respiratory system and higher risk of respiratory diseases compared to term neonates [34]. The study by Blank et al., which compared the dynamics of pulmonary aeration in late preterm and term neonates during the early adaptation period, resonates with our findings and reveals significant differences in pulmonary aeration rate and volume. Using lung ultrasound, the researchers found that during the first 24 h after birth, late preterm neonates had later and slower lung aeration compared to term neonates [35]. Our research similarly demonstrates lower lung ventilation in late preterm neonates compared to term neonates, suggesting a potential link between delayed lung ventilation and respiratory complications. Late preterm neonates are likely more susceptible to respiratory adaptation because they have not had the opportunity to practice the breathing skill that is required during the crucial transition period. The application of EIT provides a continuous picture of regional lung ventilation and allows us to understand the dynamic changes in neonatal ventilation that occur during the transition. When compared to the study conducted by McEvoy et al., which focused on assessing respiratory function in late preterm neonates using traditional spirometry methods, the EIT results provide a more nuanced perspective. McEvoy et al.’s research emphasized a decreased ratio of time to peak expiratory flow to total expiratory time (TPTEF:TE), increased respiratory resistance, and variations in tidal volume, indicating potential limitations in expiratory airflow. The differences observed in lung aeration and mechanical characteristics highlighted in this study could help to explain the findings concerning challenges among late preterm infants, as indicated by McEvoy et al. [36]. The ability of EIT to assess lung ventilation offers additional insights beyond what conventional respiratory tests can reveal, providing an alternative viewpoint on the potential factors contributing to the pulmonary susceptibility observed in late preterm infants.

The literature emphasizes the importance of understanding long-term respiratory outcomes associated with prematurity [37]. Rose et al. provided insights into the long-term respiratory consequences of late preterm birth, aligning with our findings on neonatal pulmonary ventilation dynamics. This correlation underscores the need for customized interventions and follow-up care to ensure lifelong respiratory health. Late preterm neonates, often requiring treatment for acute respiratory distress, remain at higher risk of respiratory diseases like asthma and respiratory infections later in life [38]. Pike et al. highlighted the challenges faced by neonates born between 34 and 37 weeks, emphasizing the impact of prenatal and postnatal factors on respiratory development. Their findings support the importance of identifying early markers of respiratory risk and implementing intervention strategies such as antenatal corticosteroids [39]. Natarajan et al. reported that respiratory problems in moderately or late preterm neonates can persist until mid-childhood, necessitating continuous monitoring and intervention strategies [40]. Our study adds to this knowledge by providing data on neonatal pulmonary ventilation dynamics in the postnatal period.

### Strengths and Limitations

The strength of our study is the detailed assessment of pulmonary ventilation dynamics during the early adaptation period using EIT in both late preterm and term neonates. Comparative analysis of late preterm and term neonates revealed significant differences in lung ventilation—relative stretch, center of ventilation, and tidal volume distribution—which provide important information for a better understanding of impaired early postnatal adaptation. The results have clinical implications for optimizing respiratory care strategies in this population, adapting appropriate interventions to ensure lung ventilation, and managing health outcomes. However, the study has some limitations. First of all, it is a single-center study, and this may affect the generalizability of the findings. The short-term follow-up does not allow us to assess the long-term consequences for the respiratory system.

## 5. Conclusions

Our study demonstrated differences between the dynamics of lung ventilation in late preterm and term neonates during the early adaptation period as assessed by electrical impedance tomography. Late preterm neonates showed reduced lung ventilation during the 90-min study period, suggesting a possible impairment of respiratory adaptation shortly after birth. Furthermore, the differences in the distribution of relative stretch, center of ventilation, and tidal volume between the study groups further emphasize the need for close monitoring and individualization of respiratory care. Although this study provides valuable knowledge into the early respiratory problems faced by late preterm neonates, further studies with larger samples and longer follow-up periods are needed to validate these results.

## Figures and Tables

**Table 1:** Characteristics of study and control groups.

	Study Group—Late Preterm Neonates (n = 51)		Control Group–Term Neonates (n = 45)	
	Average	Standard Deviation +/−	Average	Standard Deviation +/−
Gestational age (weeks)	35	0.58	39	0.83
Maturity index	19	1.50	26.8	1.40
Weight (g)	2756	463	3464	354
Cord blood pH	7.3	0.05	7.3	0.08
Apgar scores				
1 min	8	1.02	8	1.21
5 min	9	0.78	9	0.92

**Table 2:** Silent spaces, record I.

	Group	N	Q1	Median	Q3	Minimum Value	Maximum Value	Mann–Whitney Average Ranks	*p*-Value
Non-Dependent [%]	Preterm	51	5.69	7.00	8.90	3.88	16.98	58.10	**<0.001**
	Term	45	4.42	5.00	6.89	2.21	17.68	37.62	
Dependent [%]	Preterm	51	0.59	1.06	2.39	0.10	9.39	67.75	**<0.001**
	Term	45	0.00	0.00	0.00	0.00	9.39	26.68	

**Table 3:** Silent spaces, record II.

	Group	N	Q1	Median	Q3	Minimum Value	Maximum Value	Mann–Whitney Average Ranks	*p*-Value
Non-Dependent [%]	Preterm	45	6.28	6.91	8.50	3.97	17.70	50.47	0.071
	Term	45	4.97	6.63	7.97	1.10	18.23	40.53	
Dependent [%]	Preterm	45	0.52	1.31	2.00	0.00	5.50	61.66	**<0.001**
	Term	45	0.00	0.00	0.00	0.00	11.60	29.34	

**Table 4:** Silent spaces, record III.

	Group	N	Q1	Median	Q3	Minimum Value	Maximum Value	Mann–Whitney Average Ranks	*p*-Value
Non-Dependent [%]	Preterm	42	5.90	6.90	7.93	3.37	15.61	45.82	0.212
	Term	42	4.97	6.32	8.21	0.00	16.02	39.18	
Dependent [%]	Preterm	42	0.42	1.17	2.44	0.05	6.71	59.07	**<0.001**
	Term	42	0.00	0.00	0.00	0.00	22.10	25.93	

**Table 5:** Relative stretch, record III.

	Group	N	Q1	Median	Q3	Minimum Value	Maximum Value	Mann–Whitney Average Ranks	*p*-Value
Stretch (quartile (−))	Preterm	42	0.43	0.46	0.48	0.31	0.54	38.21	0.107
	Term	42	0.44	0.47	0.48	0.35	0.55	46.79	
Stretch (median)	Preterm	42	0.61	0.63	0.65	0.47	0.70	39.17	0.210
	Term	42	0.60	0.65	0.68	0.53	0.74	45.83	
Stretch (quartile (+))	Preterm	42	0.78	0.79	0.81	0.64	0.85	36.33	**0.021**
	Term	42	0.80	0.81	0.84	0.65	0.87	48.67	

**Table 6:** Relative tidal volume, record I.

	Group	N	Q1	Median	Q3	Minimum Value	Maximum Value	Mann–Whitney Average Ranks	*p*-Value
0.1	Preterm	51	0.79	1.01	1.18	0.41	2.12	63.25	**<0.001**
	Term	45	0.44	0.53	0.74	0.20	3.43	31.78	
0.2	Preterm	51	2.64	3.12	3.51	1.38	4.91	57.22	**0.001**
	Term	45	1.73	2.17	3.26	0.65	6.39	38.62	
0.3	Preterm	51	4.64	5.43	6.25	2.77	9.94	49.20	0.794
	Term	45	3.72	5.42	7.38	1.72	11.41	47.71	
0.4	Preterm	51	7.78	8.25	9.22	3.42	13.52	43.29	0.051
	Term	45	8.01	9.03	11.00	3.01	13.76	54.40	
0.5	Preterm	51	10.63	11.14	11.84	4.89	15.63	46.22	0.392
	Term	45	10.08	11.73	13.10	6.93	16.65	51.09	
0.6	Preterm	51	12.26	13.36	13.85	9.25	15.58	46.67	0.492
	Term	45	12.36	13.55	14.28	7.90	20.71	50.58	
0.7	Preterm	51	13.31	14.22	14.84	10.31	16.84	41.78	**0.012**
	Term	45	12.70	15.27	16.45	9.34	20.76	56.11	

**Table 7:** Relative tidal volume, record II.

	Group	N	Q1	Median	Q3	Minimum Value	Maximum Value	Mann–Whitney Average Ranks	*p*-Value
0.1	Preterm	45	0.77	0.94	1.17	0.45	3.12	57.24	**<0.001**
	Term	45	0.52	0.66	0.81	0.22	3.76	33.76	
0.2	Preterm	45	2.52	3.09	3.73	1.82	13.87	51.18	**0.039**
	Term	45	1.94	2.66	3.62	1.15	5.54	39.82	
0.3	Preterm	45	4.94	5.69	6.80	3.96	14.08	49.22	0.176
	Term	45	3.60	5.52	7.09	1.97	11.97	41.78	
0.4	Preterm	45	7.81	8.93	9.37	3.85	12.31	40.58	0.074
	Term	45	7.62	9.53	11.00	2.76	14.83	50.42	
0.5	Preterm	45	10.69	11.45	12.12	4.87	14.67	39.82	**0.039**
	Term	45	10.62	12.06	13.53	7.03	17.74	51.18	
0.6	Preterm	45	12.21	13.02	14.05	9.98	18.90	40.71	0.082
	Term	45	12.12	13.85	15.79	8.22	22.84	50.29	
0.7	Preterm	45	13.37	14.08	15.07	10.10	21.49	49.27	0.171
	Term	45	11.74	13.66	15.27	7.81	21.83	41.73	
0.8	Preterm	45	13.12	14.01	14.86	9.19	15.91	45.44	0.984
	Term	45	10.39	14.49	16.23	6.11	20.11	45.56	
0.9	Preterm	45	13.02	13.68	14.59	7.00	17.72	37.84	**0.005**
	Term	45	13.43	14.58	17.78	5.29	21.78	53.16	
1	Preterm	45	12.68	13.66	14.68	8.04	18.36	48.76	0.237
	Term	45	11.62	13.04	14.46	5.66	21.38	42.24	

**Table 8:** Relative tidal volume, record III.

	Group	N	Q1	Median	Q3	Minimum Value	Maximum Value	Mann–Whitney Average Ranks	*p*-Value
0.1	Preterm	42	0.75	0.95	1.25	0.58	1.98	53.95	**<0.001**
	Term	42	0.50	0.63	0.86	0.27	4.20	31.05	
0.2	Preterm	42	2.51	3.12	3.94	1.57	7.68	51.95	**<0.001**
	Term	42	1.82	2.22	3.25	0.20	6.29	33.05	

**Table 9:** Center of ventilation, record I.

	Group	N	Q1	Median	Q3	Minimum Value	Maximum Value	Mann–Whitney Average Ranks	*p*-Value
Right-Left [%]	Preterm	51	41.19	42.64	44.53	35.69	61.87	50.24	0.516
	Term	45	42.49	42.77	42.93	29.22	44.62	46.53	
Ventral-Dorsal [%]	Preterm	51	58.55	60.23	62.58	53.16	69.08	63.08	**<0.001**
	Term	45	55.04	55.08	55.55	54.89	64.10	31.98	

**Table 10:** Center of Ventilation, record II.

	Group	N	Q1	Median	Q3	Minimum Value	Maximum Value	Mann–Whitney Average Ranks	*p*-Value
Right-Left [%]	Preterm	45	40.77	43.48	46.92	34.79	69.60	50.44	0.073
	Term	45	42.50	42.80	42.95	21.83	54.60	40.56	
Ventral-Dorsal [%]	Preterm	45	58.35	60.02	61.67	55.66	69.61	60.42	**<0.001**
	Term	45	55.04	55.17	55.70	53.03	65.24	30.58	

**Table 11:** Center of ventilation, record III.

	Group	N	Q1	Median	Q3	Minimum Value	Maximum Value	Mann–Whitney Average Ranks	*p*-Value
Right-Left [%]	Preterm	42	41.28	43.83	46.62	31.99	57.03	46.88	0.100
	Term	42	42.50	42.78	42.95	20.69	49.18	38.12	
Ventral-Dorsal [%]	Preterm	42	57.85	59.91	61.54	51.28	64.61	55.55	**<0.001**
	Term	42	55.03	55.16	57.46	53.69	62.60	29.45	

**Table 12:** Region of interest, record I.

		Group	N	Q1	Median	Q3	Minimum Value	Maximum Value	Mann–Whitney Average Ranks	*p*-Value
**Right lung**	Ventral [%]	Preterm	51	4.62	7.58	9.23	1.39	21.59	35.55	**<0.001**
		Term	45	14.13	14.65	14.71	1.36	15.11	63.18	
	Central V [%]	Preterm	51	13.76	15.50	16.76	1.99	19.65	38.65	**<0.001**
		Term	45	16.51	16.63	16.84	11.99	25.75	59.67	
	Central D [%]	Preterm	51	19.30	22.83	25.26	12.97	29.62	59.65	**<0.001**
		Term	45	17.83	17.96	18.49	17.59	31.93	35.87	
	Dorsal [%]	Preterm	51	17.21	19.76	21.55	14.21	27.15	59.78	**<0.001**
		Term	45	16.59	16.68	17.47	16.38	24.99	35.71	
**Left lung**	Ventral [%]	Preterm	51	2.15	2.70	3.65	1.09	5.02	33.59	**<0.001**
		Term	45	4.36	4.84	4.90	0.17	4.93	65.40	
	Central V [%]	Preterm	51	6.09	6.86	7.50	2.78	12.17	45.27	0.227
		Term	45	7.06	7.15	7.18	1.63	8.52	52.16	
	Central D [%]	Preterm	51	9.25	10.44	11.61	6.41	20.21	62.69	**<0.001**
		Term	45	8.79	8.88	8.93	4.16	11.92	32.42	
	Dorsal [%]	Preterm	51	12.21	13.56	15.00	7.38	28.95	54.59	**0.023**
		Term	45	12.98	13.17	13.28	7.05	14.65	41.60	

**Table 13:** Region of interest, record II.

		Group	N	Q1	Median	Q3	Minimum Value	Maximum Value	Mann–Whitney average ranks	*p*-Value
**Right lung**	Ventral [%]	Preterm	45	5.99	7.39	10.06	0.69	14.07	31.31	**<0.001**
		Term	45	13.64	14.52	14.70	2.11	14.96	59.69	
	Central V [%]	Preterm	45	13.70	14.74	16.67	4.20	20.40	36.24	**0.001**
		Term	45	16.48	16.63	16.75	8.55	30.97	54.76	
	Central D [%]	Preterm	45	19.18	21.74	25.53	6.72	30.24	55.78	**<0.001**
		Term	45	17.88	18.00	18.93	17.63	34.04	35.22	
	Dorsal [%]	Preterm	45	17.44	19.15	21.66	8.05	28.29	55.42	**<0.001**
		Term	45	16.62	16.70	17.59	15.94	25.15	35.58	
**Left lung**	Ventral [%]	Preterm	45	2.39	3.03	4.02	0.74	6.15	33.20	**<0.001**
		Term	45	3.73	4.81	4.89	0.21	4.94	57.80	
	Central V [%]	Preterm	45	6.28	7.21	8.46	4.05	16.73	48.36	0.300
		Term	45	7.02	7.16	7.20	0.00	10.38	42.64	
	Central D [%]	Preterm	45	9.41	10.78	12.05	6.47	25.04	56.60	**<0.001**
		Term	45	8.80	8.87	9.03	0.00	17.39	34.40	
	Dorsal [%]	Preterm	45	11.85	13.39	15.72	7.78	28.16	48.44	0.285
		Term	45	13.06	13.20	13.30	0.00	21.22	42.56	

**Table 14:** Region of interest, record III.

		Group	N	Q1	Median	Q3	Minimum Value	Maximum Value	Mann–Whitney Average Ranks	*p*-Value
**Right lung**	Ventral [%]	Preterm	42	5.97	7.28	9.38	3.49	14.88	30.10	**<0.001**
		Term	42	8.44	14.49	14.73	1.42	15.13	54.90	
	Central V [%]	Preterm	42	13.40	15.21	16.75	7.35	21.80	33.74	**0.001**
		Term	42	16.44	16.63	17.13	12.89	29.98	51.26	
	Central D [%]	Preterm	42	20.51	22.63	24.93	10.86	31.59	49.76	**0.006**
		Term	42	17.85	18.06	23.02	17.65	36.67	35.24	
	Dorsal [%]	Preterm	42	17.75	19.26	21.30	9.77	24.30	51.83	**<0.001**
		Term	42	16.58	16.79	17.85	16.25	24.73	33.17	
**Left lung**	Ventral [%]	Preterm	42	2.43	3.03	3.86	1.32	6.13	33.52	**0.001**
		Term	42	2.46	4.80	4.89	0.00	4.95	51.48	
	Central V [%]	Preterm	42	6.68	7.44	8.63	3.27	12.59	47.81	**0.046**
		Term	42	6.91	7.13	7.20	0.05	10.74	37.19	
	Central D [%]	Preterm	42	9.42	11.09	12.63	5.12	18.51	53.33	**<0.001**
		Term	42	8.78	8.88	9.05	0.03	14.99	31.67	
	Dorsal [%]	Preterm	42	11.49	13.23	15.56	6.51	22.12	43.74	0.642
		Term	42	13.04	13.22	13.31	0.00	18.64	41.26	

## Data Availability

The raw data supporting the conclusions of this article will be made available by the authors upon request.
